# Improvement of absorbability, osteoconductivity, and strength of a β-tricalcium phosphate spacer for opening wedge high tibial osteotomy: clinical evaluations with 106 patients

**DOI:** 10.1186/s12891-024-07533-8

**Published:** 2024-06-05

**Authors:** Jun Yamaguchi, Eiji Kondo, Kazunori Yasuda, Jun Onodera, Koji Yabuuchi, Takuma Kaibara, Kimiaki Takami, Norimasa Iwasaki, Tomonori Yagi

**Affiliations:** 1 Knee Research Center, Yagi Orthopaedic Hospital, 1-35, Nishino-3-5, Nishi-ku, Sapporo, Hokkaido 063-0033 Japan; 2 Centre for Sports Medicine, Hokkaido University Hospital, Kita-14, Nishi-5, Kita-ku, Sapporo, Hokkaido 060-8648 Japan; 3 Department of Orthopaedic Surgery, Faculty of Medicine, Graduate School of Medicine, Hokkaido University, Kita-15 Nishi-7, Kita-ku, Sapporo, Hokkaido 060-8638 Japan; 4 Laboratory of Product Development, Olympus Termo Biomaterials Corporation, 1002-1, Shimonagakubo, Nagaizumi-cho, Shizuoka, 413-0934 Japan

**Keywords:** β-tricalcium phosphate spacer, Porosity, Absorbability, Osteoconductivity, Opening wedge, High tibial osteotomy

## Abstract

**Background:**

An ideal synthetic spacer for medial opening wedge high tibial osteotomy (MOWHTO) has not yet been developed. The authors have developed a new β-tricalcium phosphate (β-TCP) spacer with 60% porosity (N-CP60) by modifying the micro- and macro-pore structures of a conventional β-TCP spacer (CP60) that is widely used in clinical practice. The purpose of this study was to compare the absorbability, osteoconductivity, and in vivo strength of the N-CP60 spacer with those of the CP60 spacer, when used in MOWHTO.

**Methods:**

First, the porosity, diameter distribution of macro- and micropores, and compressive strength of each β-TCP block were examined using methodology of biomaterial science. Secondly, a clinical study was performed using a total of 106 patients (106 knees) with MOWHTO, who were followed up for 18 months after surgery. In these knees, the N-CP60 and CP-60 spacers were implanted into 49 tibias and 57 tibias, respectively. The absorbability and osteoconductivity were radiologically evaluated by measuring the area of the implanted spacer remaining unabsorbed and assessing with the Hemert’s score, respectively. The incidence of cracking in the implanted spacers was determined using computed radiography. Statistical comparisons were made with non-parametric tests. The significance level was set at *p* = 0.05.

**Results:**

The N-CP60 and CP60 blocks had almost the same porosity (mean, 61.0% and 58.7%, respectively). The diameter of macropores was significantly larger (*p* < 0.0001) in the N-CP60 block than in the CP60 block, while the diameter of micropores was significantly smaller (*p* = 0.019) in the N-CP60 block. The ultimate strength of the N-CP60 block (median, 36.8 MPa) was significantly greater (*p* < 0.01) than that of the CP60 block (31.6 MPa). As for the clinical evaluations, the absorption rate of the N-CP60 spacer at 18 months after implantation (mean, 48.0%) was significantly greater (*p* < 0.001) than that of the CP60 spacer (29.0%). The osteoconductivity of the N-CP60 spacer was slightly but significantly higher (*p* = 0.0408) than that of the CP60 spacer only in zone 1. The incidence of in vivo cracking of the posteriorly located N-CP60 spacer at one month (mean, 75.5%) was significantly lower (*p* = 0.0035) than that of the CP60 spacer (91.2%).

**Conclusions:**

The absorbability, osteoconductivity, and compressive strength of the new N-CP60 spacer were significantly improved by modifying the macro- and micro-pore structures, compared with the conventional CP60 spacer. The N-CP60 spacer is more clinically useful than the CP60 spacer.

**Trial registration number:**

H29-0002.

**Supplementary Information:**

The online version contains supplementary material available at 10.1186/s12891-024-07533-8.

## Background

The medial opening wedge high tibial osteotomy (MOWHTO) procedure reported by Staubli and Lobenhoffer [1, 2] is a useful surgical option for the middle-aged and elderly citizens with active lifestyles, sports enthusiasts, and workers, who are suffering from knee pain due to medial osteoarthritis (OA) of the knee. This procedure has the following advantages. First, the procedure is simple. Secondly, fibular osteotomy is not needed. Thirdly, early weight bearing can be allowed postoperatively. However, the original procedure has a few technical disadvantages [3–5]. First, this technique, in which the osteotomized tibia is fixed with a locking plate over a large vacant space in the medial tibia, is difficult to exactly achieve the preoperatively planned correction angle. Secondly, the vacant space needs a long period until it is filled with regenerated bone. To solve these disadvantages of the MOWHTO procedure, attempts have been made to develop a wedge-shaped spacer to fill the medial vacant space during surgery. It is widely agreed that autografting is the gold standard for its load-bearing, osteoconductive, osteoinductive, and osteogenic properties, and allograft spacers have been successfully used for HTO in the past 15 years, as an ideal spacer in terms of biological properties [6–15]. Moreover, not only allograft wedges are available from tissue banks, but recently some companies are distributing animal bone implants [16]. However, autografts have some problems, including donor site morbidity, while allografts have disadvantages such as the potential to provoke an immune response and the risk of disease transmission [17], although allografts risks as disease transmission are strongly reduced thanks to high quality standards and strict donor analysis and selection criteria.

The first wedge-shaped spacer was made from hydroxy apatite (HAp), because HAp had high strength and excellent osteoconductivity [4, 18–20]. However, the HAp space was barely absorbed in the tibia after implantation [18]. Beta-tricalcium phosphate (β-TCP) has attracted attention as an absorbable biomaterial [21–25]. However, the mechanical strength of previously sintered β-TCP blocks was too weak to create a spacer for MOWHTO [26]. However, Onodera et al. [18] reported that the β-TCP spacer having the porosity of approximately 60% (CP60) was strong enough to be clinically used in MOWHTO. However, they also described that the absorption rate of the CP60 spacer was only 25% at 1 year after implantation. Furthermore, the authors frequently experienced cracking in the CP60 spacer after implantation. This phenomenon shows that the strength of the CP60 spacer is insufficient. Thus, an ideal synthetic spacer for MOWHTO, which has higher absorbability, osteoconductivity, and strength in vivo, has not yet been developed [18, 27].

Recently, the authors have developed a new β-TCP (N-CP60) spacer, which has the same porosity (approximately 60%) as the CP60 spacer but different micro- and macro-pore structures from the CP60 spacer. The N-CP60 spacer is expected to have higher absorbability, osteoconductivity, and compressive strength than the CP60 spacer. However, this expectation has not been evaluated in vivo. Therefore, the present clinical study is conducted to evaluate whether the absorbability, osteoconductivity, and in vivo strength of the N-CP60 spacer implanted into the tibia are superior to those of the CP60 spacer. The purpose of this study is to test the following hypotheses: Firstly, the absorption rate of the N-CP60 spacer at 18 months after implantation may be significantly greater than that of the CP60 spacer. Secondly, the osteoconductivity of the N-CP60 spacer at 18 months after implantation may be better than in the CP60 spacer. Thirdly, the incidence of in vivo cracking of the N-CP60 spacer found at one month after implantation may be significantly lower than that of the CP60 spacer.

## Methods

### Materials

**Fig. 1:**
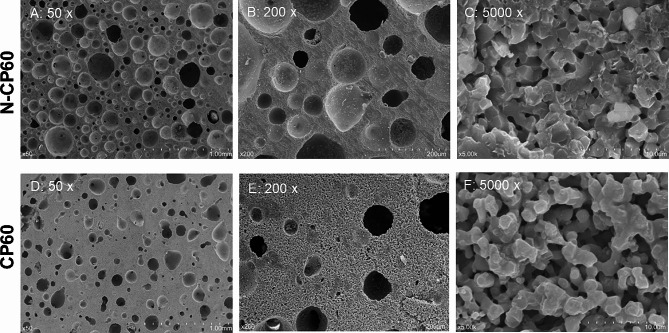
The macro- and micro-structures of each material surface, which was not embedded with resin, were observed with SEM. The surface of each material was polished and coated with gold before the observation. (**A**) N-CP60 (x50), (**B**) CP60 (x50), (**C**) N-CP60 (x5000,) (**D**) CP60 (x5000)

The CP60 block (Osferion 60®, Olympus Terumo Biomaterials Co., Tokyo, Japan) and the N-CP60 block (Osferion 60 Marvelous®, Olympus Terumo Biomaterials Co.), which were commercially available for clinical use in Japan, were used in this study (Fig. 1). The CP60 block was synthesized with a mechanochemical method as follows: CaHPO4 and CaCO3 (at a molar ratio of 2:1) were mixed with pure water in a pot mill for 24 h, creating slurry. The slurry was dried at 80 °C. The calcium-deficient HAp was converted to β-TCP by calcinations at 750 °C for 10 h. The obtained β-TCP powder was mixed with pure water and a foaming agent. After preform blocks of β-TCP were dried, they were sintered at 1050 °C for 1 h.

The N-CP60 block was manufactured using the following method that was an improvement of the above-described method to synthesize the CP60 block. Namely, first, the particle size of β-TCP powder for the N-CP60 block (approximately 0.5 micrometers in the diameter) was made to be finer than that for the CP60 block (approximately 1 micrometers). Secondly, the time for foaming in the slurry was elongated so that the number of macropores, which were greater than 10 micrometers in the diameter, was increased and the macropores were evenly distributed. Thirdly, the preforming block was sintered at 1050 °C for 3 h.

###  Measurement of porosity, pore structures, and strength

The porosity of each block sample was calculated from accurate measurements of mass and size, using the following formula: Porosity (%) = { 1 - [ ( W/3.07 ) / V ] } * 100. Here, W and V are the measured weight (gram) and volume (cm^3^), respectively, of each sample. “3.07” means the theoretical density of β-TCP (g/cm^3^). For each material, 1000 samples were evaluated.

Macropores were defined as pores having a diameter greater than 10 micrometers, according to ISO13175. Distribution of the diameter of macropores in each block sample was determined using a scanning electron microscope (SEM: SU-1510, Hitachi High-Tech Corp, Tokyo, Japan) according to previous studies [28–30]. For each material, 9 block samples were used for this evaluation. Each block was embedded with UV-curable resin (Technovit 3040, Kulzer GmbH., Wehrheim, Germany), and its polished surface was observed with the SEM. On each surface, a back-scattering electron image was taken at 3 parts which were randomly selected. In each image, the Heywood diameter of each pore was measured using the image analysis software (ImageJ, National Institutes of Health, USA).

**Fig. 2:**
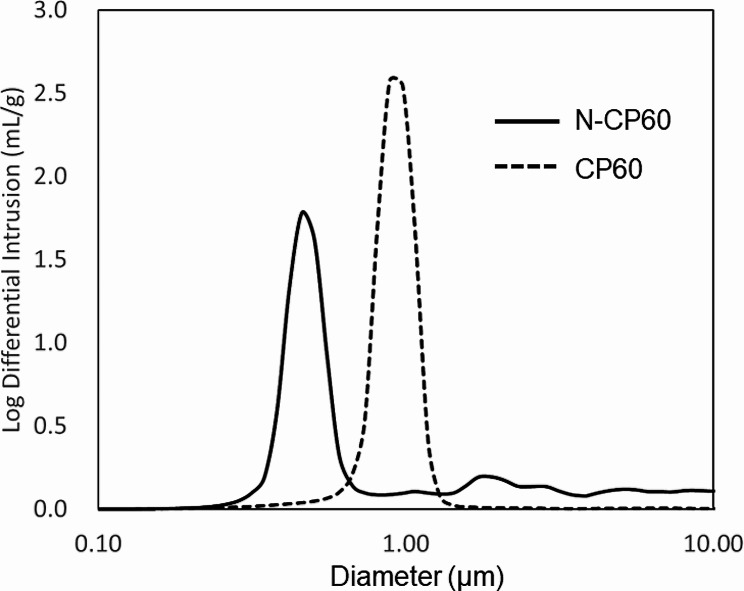
Representative curves showing distribution of the micropore diameters (< 10 micrometers), which were measured with the Mercury porosimetry. Each distribution curve has a clear peak, which shows the mode in the diameter distribution. Therefore, the mode values were statistically compared between the CP60 and N-CP60 specimens

Concerning micropores, which were defined as those pores having a diameter of less than 10 micrometers, the total volume and the mode in the diameter distribution was evaluated using a mercury porosimeter (Autopore IV 9520, Micromeritics Instrument Corp., Norcross, USA). In the mercury porosimetry, mercury was pressed into the pores in a solid surface, and the pore distribution is obtained from the relationship between the pressure applied and the volume of mercury injected. Namely, a curve of the log differential intrusion was obtained for each sample (Fig. 2). A peak of this curve shows the mode of diameter in the distribution of the micropore diameters. Five block samples were examined in each β-TCP material. The mode values were compared between the CP60 and N-CP60 blocks.

The crystalline phases of N-CP60 and CP60 were identified using an X-ray diffractometer (Ultima IV, Hitachi Tokyo, Japan). X-rays with a wavelength of 0.154 nm were used for analysis, with a scan speed of 0.5°/min.

Ultimate compressive strength of each block was determined by compressive testing using a universal testing machine (AutoGraph, Shimadzu Corp., Kyoto, Japan). Ten cubic samples (10 × 10 × 10 mm) were used according to the previous study [31]. Each cubic sample was compressed until failure at a crosshead speed of 0.5 mm/min. The ultimate strength was determined from the obtained load-deformation curve.

###  Clinical study design

A prospective comparative study was conducted in Yagi Orthopaedic Hospital between 2012 and 2020, involving a total of 128 patients (128 knees) who underwent MOWHTO with a locking plate, TomoFix® plate (Synthes GmbH, Solothurn, Switzerland) or TriS® plate (Olympus Terumo Biomaterials Co.). The following study design was accepted by the Ethical Review Board in this hospital (08/04/2012, jRCT1013230013) and informed consent was obtained from all individual participants. The inclusion criteria of this study were (1) patients who had suffered from persistent knee pain due to medial OA, after conservative treatments were applied for 3 months or more; and (2) patients who underwent a second surgery to remove the implanted plate and screws at approximately 1 year after the HTO surgery. In Japan, patients commonly wanted the metal plate to be removed from the tibia, regardless of the clinical result of the HTO surgery. As a result of the implanted hardware removal, radiological evaluations of the implanted spacer and the surrounding bone tissue could be performed without any halation effect due to the existence of metal materials. The exclusion criteria for MOWHTO surgery included (1) lateral femorotibial angle (FTA) greater than 182°; (2) a loss of knee extension greater than 10°; (3) range of knee motion less than 130°; (4) history of knee infection; (5) moderate or severe patellofemoral joint OA; (6) collateral or cruciate ligament insufficiency; (7) osteotomy correction angle over 10°.

**Fig. 3:**
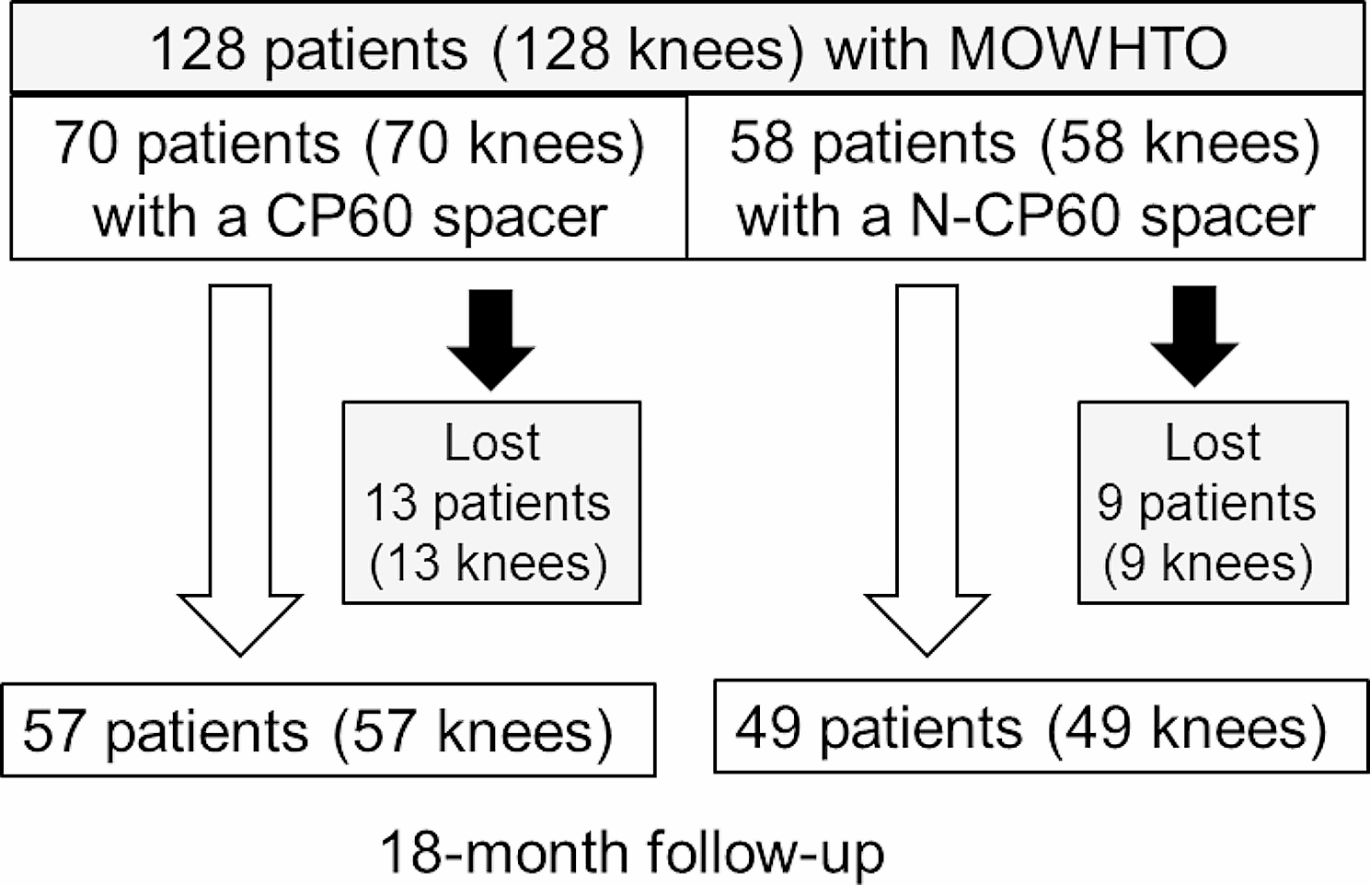
Flowchart of the patients in the 2 study groups. MOWHTO: medial opening wedge high tibial osteotomy; CP60: conventional β-TCP spacer; N-CP60: newly developed β-TCP spacer

**Fig. 4:**
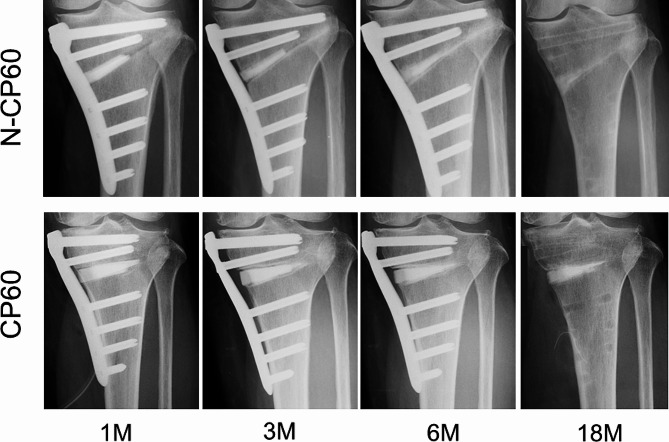
Representative cases showing the absorption and osteoconduction process of the N-CP60 and CP60 spacers. The N-CP60 case shows that the absorption rate was 86% and the osteoconductivity was evaluated as 5 points at 18 months, while the CP60 case indicated that the absorption rate was 31% and the osteoconductivity was assessed as 4 points

The first consecutive 70 knees underwent MOWHTO with the CP60 spacer between 2012 and 2017 (CP60 Group). The remaining 58 knees underwent MOWHTO with the N-CP60 spacer between 2018 and 2020 (N-CP60 Group). The operations were performed by 3 senior orthopaedic surgeons (EK, KY, and TY) who were sufficiently trained in knee surgery. Of the 128 patients, 22 patients (22 knees) were lost to follow-up. Therefore, the present study included a total of 106 patients, 57 patients in the CP60 group and 49 patients in the N-CP60 group, who were followed up in the outpatient clinic of this hospital for a period of 18 months after the surgery (Fig. 3). For each patient, radiological examinations were performed before surgery and at 1 month and 18 months after the surgery, while clinical examinations were performed before surgery and at 18 months after the surgery (Fig. 4).

###  Procedure of MOWHTO

The MOWHTO procedure was reported in detail previously [18, 32]. Briefly explained, the proximal tibia was exposed through a 7-cm medial longitudinal incision. Complete release of the distal attachment of the superficial medial collateral ligament was performed. Then, we performed an ascending biplanar osteotomy of the tibia, which consisted of an oblique HTO and a frontal plane osteotomy behind the tibial tubercle, using a thin bone saw. The oblique HTO site was then gradually opened by use of a protractor-installed spreader (Olympus Terumo Biomaterials Co.) under fluoroscopic control based on preoperative planning. During each surgery, 2 wedge-shaped spacers were created from a rectangular CP60 block or a N-CP60 block by using a circular saw system (Olympus Terumo Biomaterials Co.), and they were implanted into the anterior and posterior parts of the opening-space. Finally, the tibia was fixed with a compression locking plate by insertion of locking screws.

###  Postoperative rehabilitation

After surgery, all patients underwent postoperative management using the same rehabilitation protocol reported previously. Straight-leg raising and quadriceps sitting exercises as well as active and passive knee motion exercises were encouraged on the day after surgery. Partial weight bearing was permitted with crutches at 2 weeks after surgery. Full weight bearing was allowed at 4 weeks after surgery.

### Radiological evaluation methods

####  Alignment of the knee

In each patient, radiological evaluations were performed with computed digital radiographs (Fujifilm, Miyagi, Japan) by 2 orthopaedic surgeons (JY and KT). Anteroposterior (AP) view radiographs of the knee and the whole lower limb were taken in the single-leg standing position. Lateral and skyline view radiographs at 30° of flexion were taken in the unloaded condition. The radiological stage of OA was determined on the AP radiograph of the knee according to the Kellgren–Lawrence grading system [33]. On the AP view of the whole limb radiograph, the FTA, the point at which the weight bearing line (WBL) passed across the joint line (%MA). The FTA was defined as the angle between the axis of the femoral shaft and the axis of the tibial shaft on the fibular side. The %MA was defined as follows: A line was drawn from the center of the femoral head to the middle point of the proximal talar joint surface, and a point at which this line passed across the joint line (WBL point) was determined. The %MA was calculated as a percentage of the horizontal distance from the WBL point to the medial edge of the tibial plateau, divided by the width of the tibial plateau.

####  Absorbability of the implanted spacer

Assessment of the absorbability of each spacer was performed using the previously reported method [18] by the two authors (KT and NI). Namely, the area of the implanted spacer remaining unabsorbed was measured on the AP radiograph with the NIH Image J (National Institutes of Health, Bethesda, Maryland) immediately after surgery (A0) and at the final follow-up period (18 months after the surgery; A1). The absorption rate of each implanted spacer was calculated by the formula, (A0 - A1)/A0. First, a digital image file of a radiograph was opened by the NIH Image J. Then, for setting measurement scale, the maximum joint width of the proximal tibia was marked on the anteroposterior radiograph as a baseline. The automated threshold included only the spacer area. Finally, the area of the spacer was calculated.

####  Osteoconductivity of the implanted spacer

**Fig. 5:**
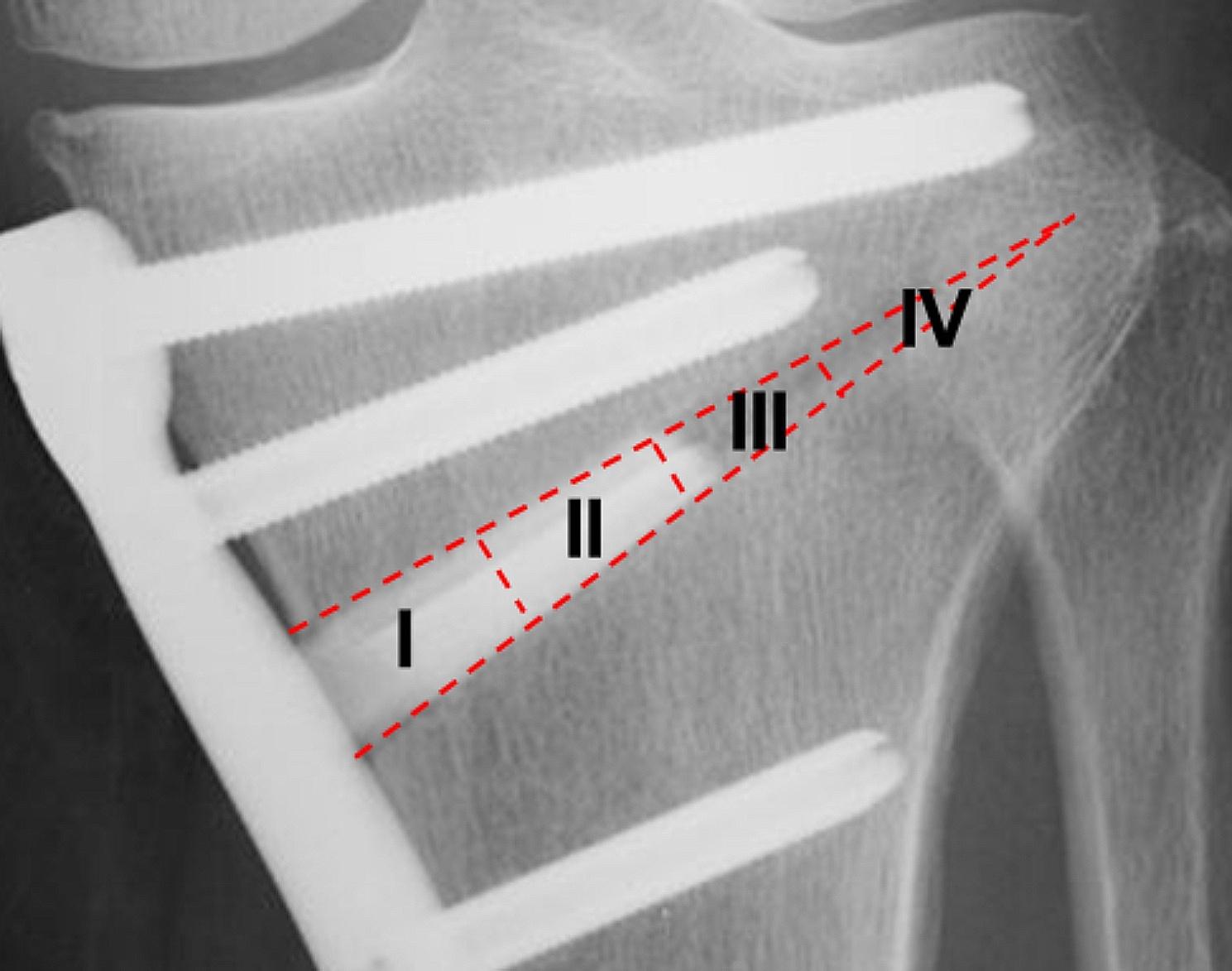
To evaluate the osteoconductivity of the implanted spacer, an elongated triangle which showed the wedge-shaped space created by open-wedge HTO was drawn on a radiograph. This elongated triangle was divided into four zones, Zone I (the most medial), II, III, and IV (the most lateral)

To evaluate the osteoconductivity of the implanted spacer, an elongated triangle, which showed the wedge-shaped space created by MOWHTO, was drawn on an AP radiograph (Fig. 5). Then, this triangle was divided into four zones [34]. The most medial zone was named ‘‘Zone I’’, and the next 3 zones were named ‘‘Zones II, III, and IV (the most lateral zone)’’, respectively. In each zone, the authors graded new bone formation in a thin gap between the bone surface and the inserted spacer surface, using the modified Hemert’s rating score [35]. Namely, 0 point was provided when an osteotomy line on the tibia was as clear as that immediately after surgery and any new bone formation was not found on either the proximal or distal surface of the spacer; 1 point was given when an osteotomy line on the tibia became unclear but a distinct lucent line was clearly visible on both the proximal and distal surface of the spacer; 2 points were given when a blurred lucent line was visible in a limited part on both the proximal and distal surface of the spacer; 3 points were given when a blurred lucent line was clearly visible on one surface of the spacer but not visible at all on the other surface; 4 points were given when a blurred lucent line was visible in a limited part on one surface of the spacer but not visible at all on the other surface; and 5 points were given when no lucent line was visible at all on either surface of the spacer. Three experienced orthopaedic surgeons (TK, JO, JY), who were blinded to all clinical information, acted as observers and independently scored each set of radiographs. Interrater reliability was evaluated by comparing the scores of the 3 observers. To calculate intra-rater consistency, one of the observers (JO) was asked to score the radiographs again at 4 weeks after the initial assessment.

####  Cracking in the implanted spacer

**Fig. 6:**
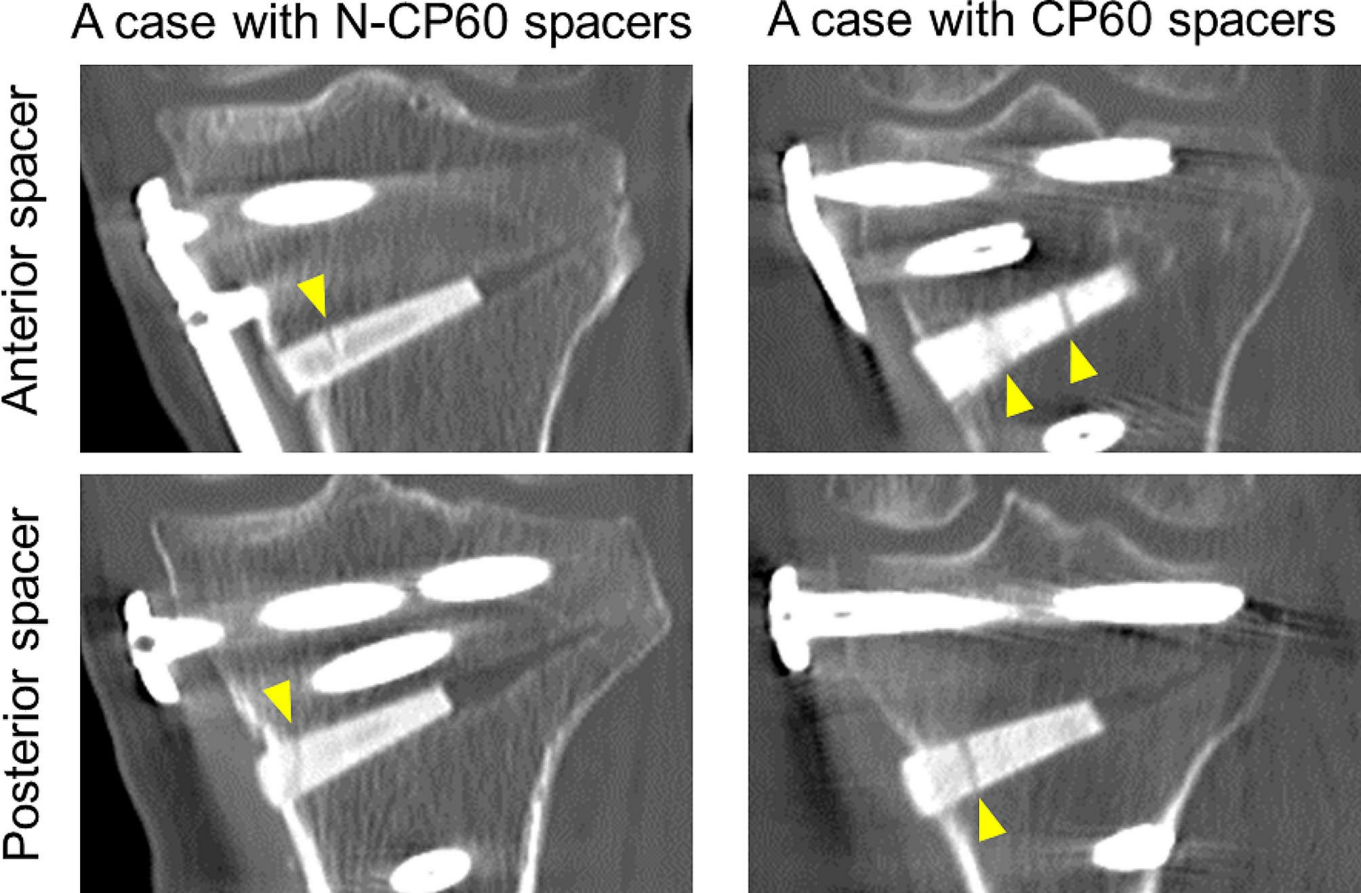
CT images of cracks (indicated with yellow arrows) in a representative case with N-CP60 or CP60 spacers. In each patient, 2 spacers were implanted anteriorly and posteriorly in the opening space of the tibia

To assess the in vivo strength of the spacer after implanted in the in vivo condition, the authors searched for cracking or collapse in the 2 spacers, which had been implanted anteriorly and posteriorly in the opening space created in the tibia, using computed tomography (CT) taken at one month after surgery (Fig. 6). The reason why the evaluation was performed at this period was that, in the authors’ experience, the cracking of the spacer commonly occurred during surgery or in the early phase after surgery, but it rarely occurred after the 1-month period. When a few thin clear lines which penetrated through the implanted spacer were found on the CT images, the spacer was defined as “cracked”. When the medial side of the implanted spacer was crushed so that the height of the spacer was reduced, the spacer was defined as “collapsed”. When cracks or collapses were not found inside the spacer, the spacer was defined as “intact”.

###  Clinical evaluations

The short-term clinical results concerning symptoms and knee functions were evaluated at the preoperative and final follow-up periods, using the Lysholm score [36] and the Japanese Orthopaedic Association (JOA) score [37, 38] which was the standard knee function scale in Japan.

###  Statistical analysis

Each parameter concerning the material characteristics was compared between the CP60 and N-CP60 blocks using the Wilcoxon rank sum test. The quadratic-weighted kappa was used to assess inter- and intra-rater reliabilities concerning the osteoconductivity evaluated with the modified Hemert’s score. To statistically compare the absorbability, the osteoconductivity, and the incidence of cracking between the CP60 and N-CP60 groups, the Mann-Whitney U test or the Fisher’s exact test was used. Calculation was made with IBM SPSS Statistics, version-23 (IBM Corp, Armonk, New York). The significance level was set at *p* = 0.05. As for an a priori power analysis for a clinical study, the sample size of 107 knees was calculated to have a power greater than 80% to test the study hypothesis.

## Results

###  Porosity, pore distribution, crystalline phases, and strength of each β-TCP

**Fig. 7:**
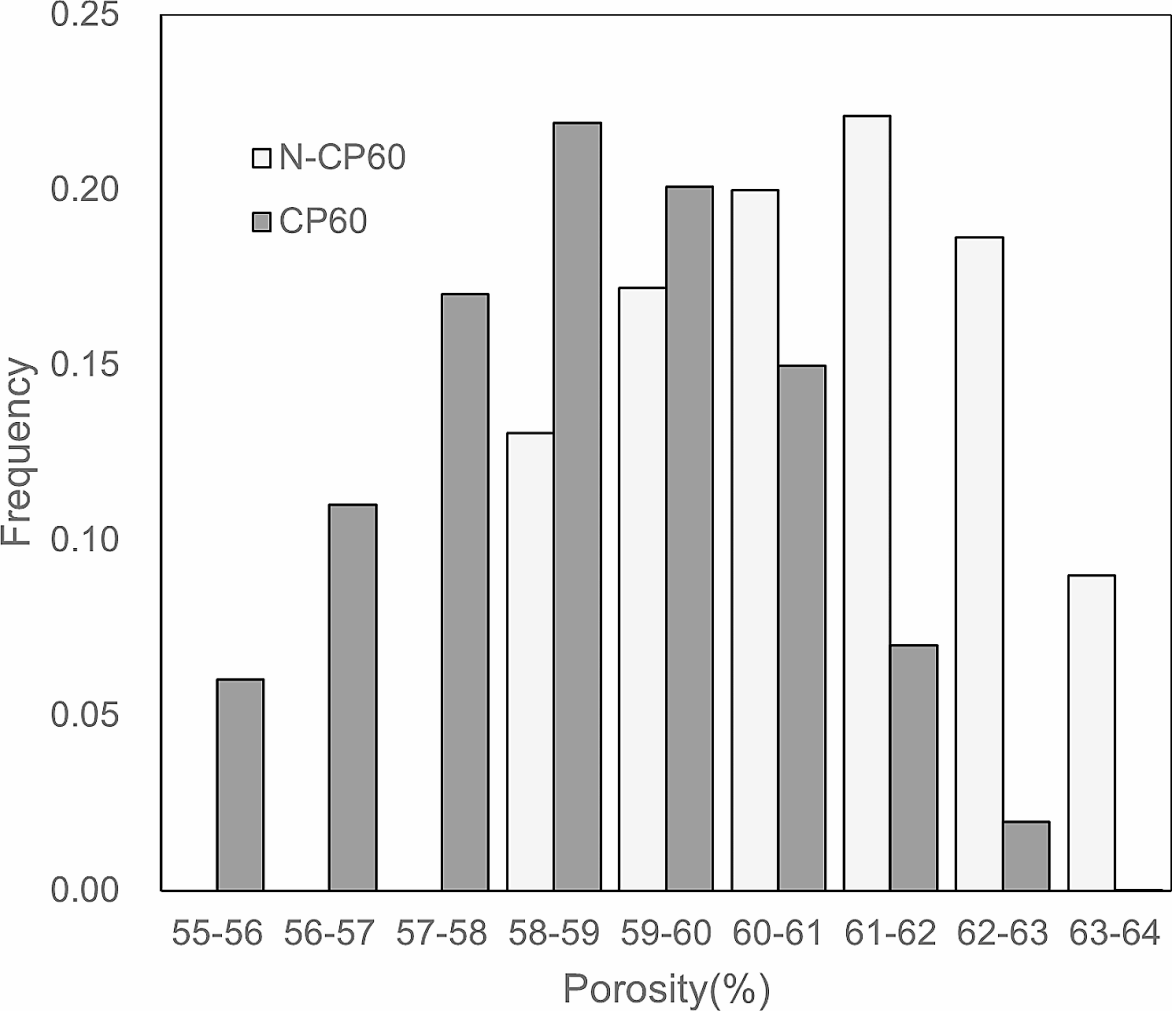
Histograms on porosity of N-CP60 and CP60 blocks. The porosity (%) of the N-CP60 block (mean ± standard deviation: 61.0 ± 1.5) was slightly but significantly (*P* < 0.0001) greater than that of the CP60 block (58.7 ± 1.7)

**Fig. 8:**
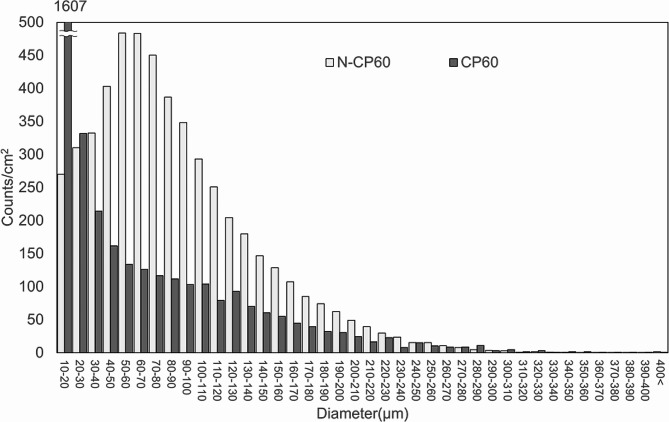
Histograms of the dimeter of macropores (> 10 μm). The dimeter distribution was significantly different between the N-CP60 and CP60 blocks (Wilcoxon rank sum test: *P* < 0.0001)

**Fig. 9:**
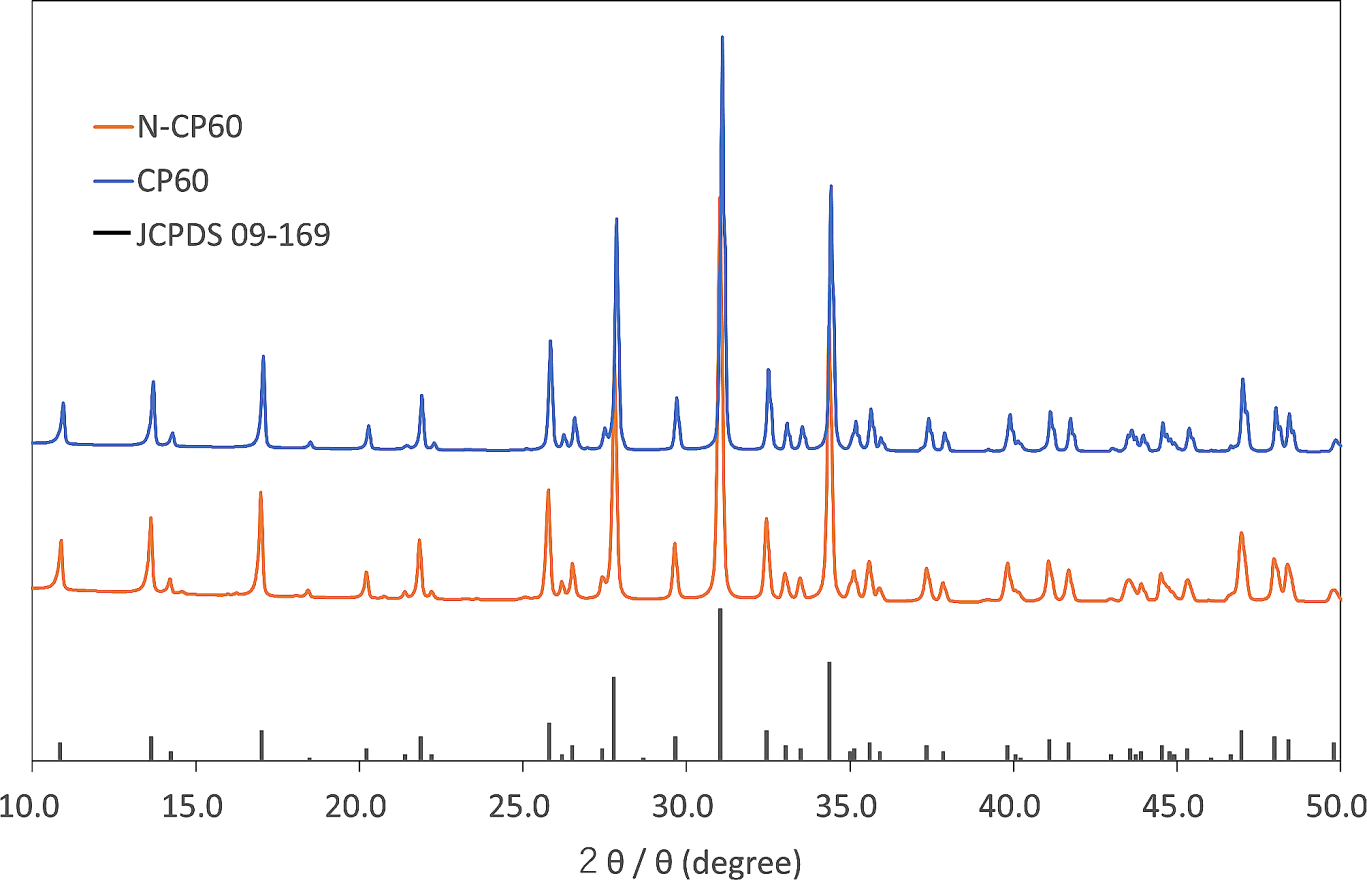
X-ray diffraction patterns of N-CP60 and CP60

**Fig. 10:**
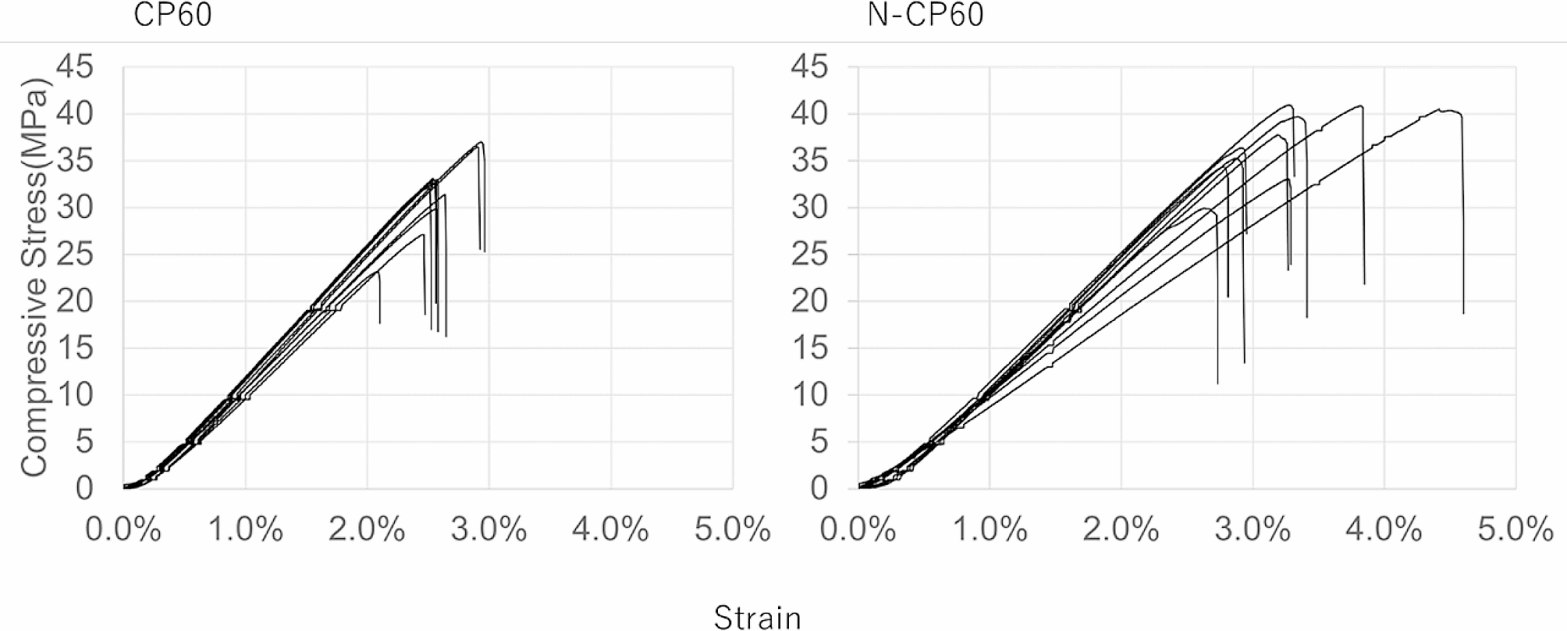
Stress-strain curves of N-CP60 and CP60

The porosity of the N-CP60 block (61.0 ± 1.5%) was slightly but significantly (*p* < 0.0001) greater than that of the CP60 block (58.7 ± 1.7%) (Table 1; Fig. 7). The mode and the mean in the diameter distribution of the macropores in the N-CP60 block (50.5 and 87.9 micrometers, respectively) were significantly greater (*p* < 0.0001) than those in the CP60 block (10.5 and 57.0 micrometers, respectively) (Table 1; Fig. 8). In the distribution of the micropore diameter (< 10 micrometers), the mode of the N-CP60 block (median, 0.58 micrometers) was significantly less (*p* = 0.019) than that of the CP60 block (0.94 micrometers) (Table 1). The macro- and micro-pores were partially interconnected under SEM evaluation. The results of X-ray diffraction measurement were shown in Fig. 9. Both of them were matched the pattern of β-TCP (JCPDS 09-169). The X-ray diffraction pattern obtained does not show any obvious HAp peaks. The stress-strain curve of compressive test of N-CP60 and CP60 was shown in Fig. 10. Mechanically, the ultimate compressive strength of the N-CP60 block (median, 37.0 MPa) was significantly greater (*p* < 0.01) than that of the CP60 block (32.4 MPa) (Table 1).

**Table 1:** Comparisons of the porosity, pore distribution, and the compressive strength between the CP60 and N-CP60 blocks

		*N*-CP60	CP60	*p*-value
Porosity (%)	Sample number	1000	1000	*< 0.0001*
	Median	61.0	58.7	
	Mean	61.0	58.7	
	SD	1.5	1.7	
	Minimum	57.0	55.0	
	Maximum	64.0	63.0	
*Macropores*: Distribution of the diameter (micrometer)	Sample number	9	9	*< 0.0001*
	Mode	50.5	10.5	
	Median	77.0	26.0	
	Mean	87.9	57.0	
	SD	53.4	62.2	
	Minimum	10.0	10.0	
	Maximum	804.6	480.3	
*Micropores*: Total volume (mL/g)	Sample number	5	5	*0.550*
	Median	0.41	0.41	
	Mean	0.38	0.40	
	SD	0.05	0.02	
	Minimum	0.30	0.37	
	Maximum	0.41	0.43	
*Micropores*: Mode in the diameter distribution (micrometer)	Sample number	5	5	*0.019*
	Median	0.58	0.94	
	Mean	0.56	0.93	
	SD	0.05	0.05	
	Minimum	0.50	0.86	
	Maximum	0.60	1.00	
Compressive strength (MPa)	Sample number	10	10	*< 0.01*
	Median	37.0	32.4	
	Mean	36.8	31.6	
	SD	3.7	4.1	
	Minimum	37.0	23.1	
	Maximum	40.9	29.9	

###  Clinical demographics

There were 33 women and 16 men in the N-CP60 group, and 46 women and 11 men in the CP60 group (Table 2). The follow-up rate was 81.4% in the CP60 group and 84.5% in the N-CP60 group, with no significant difference. The other baseline demographic and characteristic data of the patients were shown in Table 2. Preoperatively, there were no significant differences in the age, sex, body weight, body mass index between the CP60 and N-CP60 groups, although significant differences were found in the height (*p* = 0.030), the bone mineral density (*p* = 0.014), the OA grade (*p* < 0.001), FTA (*p* = 0.048), and %MA (*p* = 0.049). Intraoperatively, there were no significant differences in the correction angle by the HTO or the additional treatments with the HTO.

**Table 2:** Comparisons of the baseline demographics and characteristics of the patients between the N-CP60 and CP60 groups. Bone mineral density was shown as the rate (%) to the young adult mean (YAM). FTA: lateral femorotibial angle, MA: mechanical axis, MPTA: medial-proximal tibial angle, Corrected angle: the tibial angle corrected by HTO, OAT: osteochondral autograft transfer. The continuous data are reported as “the mean ± the standard deviation”. The discrete data are shown as “the number of the knees (%)”. The *P* value shows the result of comparison between the 2 groups

Period	Items	*N*-CP60	CP60	*P* value
	Knees (Patients)	49 (49)	57 (57)	
Preoperative	Male / Female	16 / 33	11 / 46	0.116
	Age (years)	60.4 ± 8.6	59.9 ± 8.6	*0.724*
	Height (cm)	162.1 ± 10.7	157.5 ± 7.2	*0.030*
	Body weight (kg)	67.9 ± 15.7	63.3 ± 10.8	*0.221*
	Body mass index (kg/m²)	25.7 ± 5.1	25.5 ± 3.6	*0.637*
	Bone mineral density (%)	87.2 ± 10.1	93.1 ± 11.3	*0.014*
Preoperative	OA Grade 1 (knees)	14 (28.5%)	2 (3.5%)	*< 0.001*
(Radiological)	Grade 2	23 (46.9%)	21 (36.8%)	
	Grade 3	11 (22.4%)	29 (50.9%)	
	Grade 4	1 (2.0%)	5 (8.8%)	
	Preoperative FTA (°)	178.3 ± 1.8	177.3 ± 2.3	*0.048*
	Preoperative %MA (%)	29.1 ± 9.7	33.6 ± 10.4	*0.049*
Intraoperative	Corrected angle (°)	8.2 ± 1.0	8.3 ± 0.9	*0.617*
	medial-meniscectomy (knees)	20 (40.8%)	28 (49.1%)	*0.437*
	OATS (knees)	1 (2.0%)	4 (7.0%)	*0.370*

###  Absorbability and osteoconductivity

The absorption rate was significantly greater (*p* < 0.0001) in the N-CP60 group (mean, 48.0 ± 18.1%) than in the CP60 group (29.0 ± 15.4%) (Table 3).

Concerning the osteoconductivity, the modified Hemert’s score in zone 1 showed 3 points in 1 knee, 4 points in 1 knee, and 5 points in 47 knees in the N-CP60 group, while it revealed 2 points in 4 knees, 4 points in 4 knees, and 5 points in 48 knees in the CP60 group (Table 3). There was a slight but significant difference between the 2 groups concerning the osteoconductivity in zone 1 (*p* = 0.0408). However, there were no significant differences between the 2 groups in the other zones, as all scores for both groups were given full marks. As for the intrarater reliability of the scoring with the modified Hemert’s score, the weighted kappa was 0.936 (95% CI, 0.881–0.990), indicating strong agreement. Regarding the interrater reliability, the weighted kappa was 0.900 (95% CI, 0.826–0.973) between raters 1 and 2; 0.855 (95% CI, 0.813–0.956) between raters 2 and 3; and 0.792 (95% CI, 0.687–0.897) between raters 3 and 1, all indicating strong agreement.

**Table 3:** Comparisons of the absorbability, osteoconductivity, and incidence of cracking of the implanted spacers between the CP60 and N-CP60 groups. The absorbability was shown as the absorption rate. The osteoconductivity was evaluated with the modified van Hemert’s score. The incidence of cracking was an indicator related to the strength of the spacer implanted in the in vivo condition

Evaluations	*N*-CP60 (49 knees)	CP60 (57 knees)	*P*-value
Absorption rate (%)	48.0 ± 18.1	29.0 ± 15.4	*< 0.0001*
Modified Hemert’s score in Zone1			*0.0408*
Point 2 Point 3 Point 4 Point 5	0 knees 1 1 47	4 knees 0 4 48	
Incidence of cracking (posterior spacer)			*0.0350*
Cracking (+): knees (incidence, %)	37 (75.5%)	52 (91.2%)	
Cracking (-)	12 (24.5%)	5 (8.8%)	
Incidence of cracking (anterior spacer)			*0.0011*
Cracking (+): knees (incidence, %)	9 (18.3%)	28 (49.1%)	
Cracking (-)	40 (81.6%)	29 (50.9%)	

###  Incidence of cracking in the implanted spacers

Concerning the posteriorly implanted spacer, the incidence of cracking was significantly lower (*p* = 0.0350) in the N-CP60 group (75.5%) than in the CP60 group (91.2%) (Table 3). Regarding the anteriorly implanted spacer, the incidence of cracking was significantly lower (*p* = 0.0011) in the N-CP60 group (18.3%) than in the CP60 group (49.1%) (Table 3). However, collapse of the spacer was not found out in either group.

###  Clinical and functional results of the HTO

Clinical and functional evaluations of the patients before and after the HTO surgery are shown in Table 4. There were no significant differences in each clinical result between the N-CP60 and CP60 groups, preoperatively or postoperatively. In each group, the JOA and Lysholm scores were significantly improved at the final follow-up period (*p* < 0.001), while there were no significant differences in the range of knee motion.

**Table 4:** Comparisons of the clinical and functional evaluations of the patients between the N-CP60 and CP60 groups. JOA: Japanese Orthopaedic Association. The data are shown as “the mean ± the standard deviation”. The *P* values show the results of comparing each item between the 2 groups. Additionally, the effect of the surgery in each group is evaluated by comparing the pre- and postoperative data on each item. The *P* value of this comparison is shown using the following superscript: *p < 0.001

Time of evaluation	Items evaluated	*N*-CP60	CP60	*P* value
Number of knees		49	57	
Preoperative	Knee extension (°)	-2.3 ± 3.0	-4.1 ± 5.0	*0.131*
	Knee flexion (°)	140.5 ± 14.2	140.2 ± 9.6	*0.264*
	JOA score (points)	74.7 ± 14.3	70.7 ± 11.3	*0.142*
	Lysholm score (points)	71.6 ± 13.8	67.0 ± 14.4	*0.097*
Postoperative	Knee extension (°)	-2.0 ± 2.2	-2.4 ± 3.5	*0.477*
	Knee flexion (°)	144.1 ± 10.2	144.0 ± 7.7	*0.397*
	JOA score (points)	*93.3 ± 5.5	*92.1 ± 7.2	*0.533*
	Lysholm score (points)	*94.1 ± 7.1	*91.6 ± 8.7	*0.112*

## Discussion

The most important findings of the present study were the followings: First, the absorption rate of the N-CP60 spacer at 18 months after implantation was significantly greater than that of the CP60 spacer (mean, 48.0% versus 29.0%). Secondly, the incidence of in vivo cracking of the posteriorly located N-CP60 spacer at one month after implantation was significantly lower than that of the CP60 spacer (mean, 75.5% versus 91.2%). Thirdly, the osteoconductivity of the N-CP60 spacer was slightly but significantly higher than that of the CP60 spacer only in zone 1, while there were no significant differences between the 2 spacers in the other zones. To explain causes of the superiority of the N-CP60 spacer, the present study clarified the macro- and micro-pore structures of the N-CP60 and CP60 blocks. First, the N-CP60 and CP60 blocks had similar porosity of approximately 60% (61.0% and 58.7%, respectively), while the difference was statistically significant because of the numerous samples. Secondly, the diameter of macropores present in the N-CP60 block was significantly larger than that of the CP60 block, while the micropore diameter in the N-CP60 block was significantly less than that in the CP60 block. Thirdly, the ultimate compressive strength of the N-CP60 block (median, 37.0 MPa) was significantly greater than that of the CP60 block (32.4 MPa). These results suggested that the absorbability, osteoconductivity, and in vivo strength of the N-CP60 spacer were significantly improved by alteration in the macro- and micro-pore structures in the densified β-TCP, compared with the CP60 spacer.

The authors now consider whether the N-CP60 spacer or the CP60 spacer is more useful clinically. Commonly, an ideal spacer for MOWHTO has been considered to have higher absorbability, osteoconductivity, and a similar compressive strength to that of the host bone in vivo [18, 26]. Namely, the strength is necessary to maintain the planned open space without crushing during and after surgery. The osteoconductivity is needed to shorten the period taken until the space is filled with regenerated bone. The high absorbability of the spacer is an important characteristic, if a revision or conversion surgery would be needed due to infection, pseudoarthrosis, unaccepted malalignment, or recurrent pain. Namely, the larger the mass of artificial material remaining in the bone, the greater the obstacle to a necessary surgery. The present study showed that the absorbability, osteoconductivity, and in vivo strength of the N-CP60 spacer were significantly superior to those of the CP60 spacer. However, there were no significant differences in the short-term clinical outcomes between the two groups. This fact suggests that both types of spacers are clinically useful. However, the incidence of in vivo cracking of the posteriorly located N-CP60 spacer at one month after implantation was significantly lower than that of the CP60 spacer. The radiological outcome presented a higher osteointegration for the N-CP60 wedge at 18 months after implantation, therefore demonstrating its superiority. In addition, the absorption rate of the N-CP60 spacer was about twice that of the CP60 spacer. Therefore, the expected complete resorption time for CP60 is much higher than for N-CP60. From the point of view of dealing with the risk of contingencies, the authors consider that the N-CP60 spacer is more clinically useful than the CP60 spacer.

Based on the fundamental data on the pore distribution clarified in the present study, the authors explain the causes as to why the absorption rate of the N-CP60 spacer was significantly higher than that of the CP60 spacer in the present study. Tanaka et al. [39] reported that the absorption rate of TCP blocks depends on their porosity. In the present study, the mean porosity of the N-CP60 block (61.0%) was significantly higher than that of the CP60 block (58.7%). This fact may explain in part why the absorption rate of the N-CP60 spacer was higher. However, because the difference in the porosity between the N-CP60 and CP60 blocks was only 2.3%, it cannot completely explain the large difference in the absorption rates (48.0% versus 29.0%). It has been suggested that the mechanism of bioceramic resorption involves two processes, which are solution-mediated and cell-mediated disintegrations [40, 41]. Solution-mediated disintegration is associated with the composition of the material itself as well as the surrounding environment [42, 43]. Cell-mediated disintegration is mainly caused by osteoclasts [44]. Histological assessment [45] revealed that numerous osteoclasts were present on the surface of the TCP. The main difference between N-CP60 and CP60 concerns macro and micro porosities. These devices had similar quantities of material but different spatial distribution of the material in the volume. We believe that this different spatial distribution is the key factor for the higher absorbability and osteointegration, maybe because the N-CP60 resembles the trabecular bone better than CP60.

Therefore, consideration is needed about effects of the osteoclasts, because many researchers reported that the cell-mediated disintegration by osteoclasts plays a main role in the in vivo resorption of the β-TCP [39, 44–46]. First, it is well known that osteoclasts are large cells having a diameter of approximately 100 micrometers, and that they can enter into macropores with diameters of several tens of micrometers by deformation. In other words, osteoclasts cannot enter in macropores with a diameter less than that. In the present study, the diameter of macropores present in the N-CP60 block (mean 87.9 μm, median 77.0 μm, mode 50.5 μm) was significantly larger than that of the CP60 block (mean 57.0, median 26.0, mode 10.5) (Fig. 6; Table 2). This means that the number of macropores large enough for osteoclasts to enter was significantly greater on the N-CP60 block than that on the CP60 block. This factor might contribute to the increased absorption in the N-CP60 spacer. Secondly, attention should be paid to the fact that micropore structures on the wall surfaces of the macropores can activate the osteoclasts [47]. Duan et al. [48] reported that the absorption rate of β-TCP with micropores smaller than 1 micron was significantly higher than that of β-TCP with micropores greater than that. In addition, Davison et al. [49] suggested that the TCP surface made of pore structures less than 1 micron promotes osteoclast activity. In the present study, the mode in the diameter distribution of the micropores in the N-CP60 blocks averaged 0.56 micrometers and that in the CP60 blocks averaged 0.93 micrometers, showing a significant difference. There is a high possibility that this difference in the micropore distribution between the N-CP60 and CP60 spacers resulted in a significant difference in the absorption rates via the difference of osteoclast activation. Thus, it is considered that the N-CP60 spacer has significantly more macropores that can be penetrated by osteoclasts than the CP60 spacer, and that the inner wall surfaces of the macropores in the N-CP60 spacer have the micropore structure that can activate osteoclasts to a significantly higher degree than the CP60 spacer. Thus, the macro-porosity allows the osteoclasts activity in a better manner, and facilitates the new vessels formation as well.

In the previous literature, Tanaka et al. [26] reported that β-TCP blocks with 60% porosity implanted in medial opening-wedge HTO were almost completely absorbed within 3.5 years. Altermatt et al. [21] reported that most implanted β-TCP block with 60% porosity remains for at least 7 years in calcaneal bone defects. However, these reports do not contain details of the pore structures of the β-TCP blocks used. Therefore, the previous data on absorbability cannot be directly compared to the results in the present study.

Osteoconductivity of the N-CP60 block was expected to be higher than that of the CP60 block due to the following reasons. First, the osteoconductivity of β-TCP commonly correlates with the absorbability, as the osteoclast activity results in induction of osteogenesis. Secondly, as discussed in the above-described paragraph, the number of macropores large enough for osteoclasts to migrate and attach was significantly greater on the N-CP60 block than that on the CP60 block. Thirdly, Feng et al. [50] reported that, in progression of osteogenesis, blood vessels cannot penetrate pores less than 400 μm in diameter, and the present study showed that the number of macropores with diameters greater than 400 μm was higher in the N-CP60 block (5.45 /cm2) than in the CP60 block (0.79 /cm2). In the present study, although the Hermer’s score showed that the osteoconductivity of the N-CP60 spacer was slightly but significantly higher (*p* = 0.0408) than that of the CP60 spacer only in zone 1, there were no significant differences between the 2 spacers in the other zones. The reasons for the lack of absolute significance in the results are considered as follows. It is clinically known that the proximal tibia, where the spacers were implanted, is one of sites of the most vigorous bone regeneration in the human body. This may be the reason why the Hermer’s score was perfect even around the CP60 spacer in all zones except for zone 1. Therefore, it is considered that, if the N-CP60 spacer fundamentally had higher osteoconductivity than the CP60 spacer, it might be difficult to detect a statistical significance in the proximal tibia.

In the present study, the incidence of in vivo cracking after implantation was significantly lower in the N-CP60 spacer than in the CP60 spacer at 1 month after implantation, although collapse of the spacer was not found in each spacer. This result may reflect the fact that the initial compression strength of the N-CP60 block (mean, 36.8 MPa) was significantly higher than that of the CP60 block (mean, 31.6 MPa). The causes why the initial strength of the N-CP60 blocks was significantly higher than that of the CP60 blocks despite having almost the same porosity (61.0% versus 58.7%) can be explained as follows: It is well known that the initial strength of a β-TCP block is determined by that of the weakest part in all β-TCP columns, where the first destruction occurs and spreads to the surrounding area [51]. Therefore, one common strategy to increase the strength of the β-TCP block is to increase the mechanical strength and the cross-sectional area of the weakest β-TCP column. The N-CP60 block used in this study was improved according to this strategy. First, to increase the mechanical strength of the β-TCP columns, the concentration of the slurry was increased by reduction (approximately one-tenth) of the particle size of the raw TCP powder, and the degree of sintering between the particles was increased by firing at a higher temperature for a longer time. Secondly, to increase the cross-sectional area of the β-TCP columns, the slurry was uniformly foamed using a specially developed machine. Consequently, the diameter variation of the β-TCP columns in the N-CP60 block (SD 53.4) was reduced, compared with the CP60 block (SD 62.2). Thus, it is considered that these two improvements resulted in a 20–30% increase in the compressive strength of the N-CP60 block compared to the CP60 block, despite a slight increase in the average porosity.

There are some limitations in this clinical study. First, when describing the mechanism of resorption of the artificial bone discussed in this study, we compared the results with previously reported in vitro results, but we did not have data on the in vitro resorption of the artificial bone used in this study. Secondly, there were slight but significant differences concerning a part of the preoperative demographic data, such as height, bone mineral density (YAM > 80 in both groups), OA grade, FTA, %MA, between the N-CP60 group and the CP60 group. Therefore, there is a possibility that those differences might affect the outcomes. However, it is commonly considered that those factors rarely influence the absorbability, osteoconductivity, and in vivo strength of the implanted β-TCP. Thirdly, the follow-up period averaged 18 months. Therefore, the authors could not refer to differences in the longer-term result of the absorbability between the 2 spacers. Fourthy, we performed only radiological evaluations to evaluate absorbability, osteoconductivity, and the in vivo strength, although radiological examinations are most fundamental in the clinical study. Further long-term follow-up evaluations with multidisciplinary methods are needed to confirm the result that the N-CP60 spacer is superior to the CP-60 spacer concerning the absorbability and the in vivo strength. Beyond these limitations, however, the authors believe that this study has added new scientific information to the current knowledge of absorbable spacers for MOWHTO.

## Conclusions

The absorbability, osteoconductivity, and compressive strength of the new N-CP60 spacer were significantly improved by modifying the macro- and micro-pore structures, compared with the conventional CP60 spacer. The N-CP60 spacer is more clinically useful than the CP60 spacer.

### Electronic supplementary material

Below is the link to the electronic supplementary material.

Supplementary Material 1

## Data Availability

The datasets used and analyzed in the current study are available from the corresponding author on reasonable request.

## Abbreviations

A-P: Anteriposterior
β-TCP: β-tricalcium phosphate
CT: computed tomography
MOWHTO: medial open-wedge high tibial osteotomy
HTO: high tibial osteotomy
Hap: hydroxy apatite
CP60: the β-TCP having the porosity of approximately 60%
N-CP60: a new β-TCP
FTA: femorotibial angle
MA: mechanical axis
MPTA: medial-proximal tibial angle
WBL: weight bearing line
OAT: osteochondral autograft transfer
JOA: Japanese Orthopaedic Association
YAM: young adult mean

## References

[1] Staubli AE, De Simoni C, Babst R (2003). TomoFix: a new LCP-concept for open wedge osteotomy of the medial proximal tibia–early results in 92 cases. Injury.

[2] Lobenhoffer P, Agneskirchner JD (2003). Improvements in surgical technique of valgus high tibial osteotomy. Knee Surg Sports Traumatol Arthrosc.

[3] Amendola A, Panarella L (2005). High tibial osteotomy for the treatment of unicompartmental arthritis of the knee. Orthop Clin North Am.

[4] Koshino T, Murase T, Saito T (2003). Medial opening-wedge high tibial osteotomy with use of porous hydroxyapatite to treat medial compartment osteoarthritis of the knee. J Bone Joint Surg Am.

[5] Pape D, Kohn D, Van Giffen N (2013). Differences in fixation stability between spacer plate and plate fixator following high tibial osteotomy. Knee Surg Sports Traumatol Arthrosc.

[6] Finkemeier CG (2002). Bone-grafting and bone-graft substitutes. J Bone Joint Surg Am.

[7] Spahn G, Wittig R (2002). Primary stability of various implants in tibial opening wedge osteotomy: a biomechanical study. J Orthop Sci.

[8] Amendola A, Fowler PJ, Litchfield R (2004). Opening wedge high tibial osteotomy using a novel technique: early results and complications. J Knee Surg.

[9] Chazono M, Tanaka T, Komaki H (2004). Bone formation and bioresorption after implantation of injectable beta-tricalcium phosphate granules–hyaluronate complex in rabbit bone defects. J Biomed Mater Res A.

[10] Yacobucci GN, Cocking MR (2008). Union of medial opening-wedge high tibial osteotomy using a corticocancellous proximal tibial wedge allograft. Am J Sports Med.

[11] Gouin F, Yaouanc F, Waast D (2010). Open wedge high tibial osteotomies: calcium-phosphate ceramic spacer versus autologous bonegraft. Orthop Traumatol Surg Res.

[12] Lee SC, Jung KA, Nam CH (2010). The short-term follow-up results of open wedge high tibial osteotomy with using an Aescula open wedge plate and an allogenic bone graft: the minimum 1-year follow-up results. Clin Orthop Surg.

[13] Lash NJ, Feller JA, Batty LM (2015). Bone grafts and bone substitutes for opening-wedge osteotomies of the knee: a systematic review. Arthroscopy.

[14] Haghpanah B, Kaseb MH, Espandar R (2021). No difference in union and recurrence rate between iliac crest autograft versus allograft following medial opening wedge high tibial osteotomy: a randomized controlled trial. Knee Surg Sports Traumatol Arthrosc.

[15] Zaffagnini S, Dal Fabbro G, Lucidi GA (2023). Personalised opening wedge high tibial osteotomy with patient-specific plates and instrumentation accurately controls coronal correction and posterior slope: results from a prospective first case series. Knee.

[16] Grottoli CF, Cingolani A, Zambon F (2019). Simulated performance of a xenohybrid bone graft (SmartBone®) in the treatment of acetabular prosthetic peconstruction. J Funct Biomater.

[17] Bauer TW, Muschler GF (2000). Bone graft materials. An overview of the basic science. Clin Orthop Relat Res.

[18] Onodera J, Kondo E, Omizu N (2014). Beta-tricalcium phosphate shows superior absorption rate and osteoconductivity compared to hydroxyapatite in open-wedge high tibial osteotomy. Knee Surg Sports Traumatol Arthrosc.

[19] Smith J, Wilson A, Thomas N (2013). Osteotomy around the knee: evolution, principles and results. Knee Surg Sports Traumatol Arthrosc.

[20] Takeuchi R, Ishikawa H, Aratake M (2009). Medial opening wedge high tibial osteotomy with early full weight bearing. Arthroscopy.

[21] Altermatt S, Schwöbel M, Pochon J (1992). Operative treatment of solitary bone cysts with tricalcium phosphate ceramic. A 1 to 7 year follow-up. Eur J Pediatr Surg.

[22] .Chatterjea A, Van Der Stok J, Danoux CB (2014). Inflammatory response and bone healing capacity of two porous calcium phosphate ceramics in critical size cortical bone defects. J Biomed Mater Res A.

[23] Dorozhkin SV (2010). Bioceramics of calcium orthophosphates. Biomaterials.

[24] Yuan H, Fernandes H, Habibovic P (2010). Osteoinductive ceramics as a synthetic alternative to autologous bone grafting. Proc Natl Acad Sci USA.

[25] Hwang JW, Park JS, Lee JS (2012). Comparative evaluation of three calcium phosphate synthetic block bone graft materials for bone regeneration in rabbit calvaria. J Biomed Mater Res B Appl Biomater.

[26] Tanaka T, Komaki H, Chazono M (2017). Basic research and clinical application of beta-tricalcium phosphate (β-TCP). Morphologie.

[27] Ozalay M, Sahin O, Akpinar S (2009). Remodeling potentials of biphasic calcium phosphate granules in open wedge high tibial osteotomy. Arch Orthop Trauma Surg.

[28] Ribeiro GBM, Trommer RM, dos Santos LA (2011). Novel method to produce β-TCP scaffolds. Mater lett.

[29] Tricoteaux A, Rguiti E, Chicot D (2011). Influence of porosity on the mechanical properties of microporous β-TCP bioceramics by usual and instrumented Vickers microindentation. J Eur Ceram Soc.

[30] Putri TS, Hayashi K, Ishikawa K (2020). Bone regeneration using β-tricalcium phosphate (β-TCP) block with interconnected pores made by setting reaction of β-TCP granules. J Biomed Mater Res A.

[31] Yamadi S, Kobayashi S (2009). Effects of strain rate on the mechanical properties of tricalcium phosphate/poly(L: -lactide) composites. J Mater Sci Mater Med.

[32] Yabuuchi K, Kondo E, Onodera J (2020). Clinical outcomes and complications during and after medial open-wedge high tibial osteotomy using a locking plate: a 3- to 7-year follow-up study. Orthop J Sports Med.

[33] Kellgren JH, Lawrence JS (1957). Radiological assessment of osteo-arthrosis. Ann Rheum Dis.

[34] Brosset T, Pasquier G, Migaud H (2011). Opening wedge high tibial osteotomy performed without filling the defect but with locking plate fixation (TomoFix™) and early weight-bearing: prospective evaluation of bone union, precision and maintenance of correction in 51 cases. Orthop Traumatol Surg Res.

[35] van Hemert WL, Willems K, Anderson PG (2004). Tricalcium phosphate granules or rigid wedge preforms in open wedge high tibial osteotomy: a radiological study with a new evaluation system. Knee.

[36] Lysholm J, Gillquist J (1982). Evaluation of knee ligament surgery results with special emphasis on use of a scoring scale. Am J Sports Med.

[37] Aoki Y, Yasuda K, Mikami S (2006). Inverted V-shaped high tibial osteotomy compared with closing-wedge high tibial osteotomy for osteoarthritis of the knee: ten-year follow-up result. J Bone Joint Surg Br.

[38] Yasuda K, Majima T, Tsuchida T (1992). A ten-to 15-year follow-up observation of high tibial osteotomy in medial compartment osteoarthrosis. Clin Orthop Relat Res.

[39] Tanaka T, Kumagae Y, Chazono M (2015). A novel evaluation system to monitor bone formation and β-tricalcium phosphate resorption in opening wedge high tibial osteotomy. Knee Surg Sports Traumatol Arthrosc.

[40] Fujita R, Yokoyama A, Kawasaki T (2003). Bone augmentation osteogenesis using hydroxyapatite and beta-tricalcium phosphate blocks. J Oral Maxillofac Surg.

[41] Chazono M, Tanaka T, Kitasato S (2008). Electron microscopic study on bone formation and bioresorption after implantation of beta-tricalcium phosphate in rabbit models. J Orthop Sci.

[42] Juhasz JA, Best SM, Auffret AD (2008). Biological control of apatite growth in simulated body fluid and human blood serum. J Mater Sci Mater Med.

[43] Kuroda K, Okido M (2012). Hydroxyapatite coating of titanium implants using hydroprocessing and evaluation of their osteoconductivity. Bioinorg Chem Appl.

[44] Wenisch S, Stahl JP, Horas U (2003). In vivo mechanisms of hydroxyapatite ceramic degradation by osteoclasts: fine structural microscopy. J Biomed Mater Res A.

[45] Yamasaki N, Hirao M, Nanno K (2009). A comparative assessment of synthetic ceramic bone substitutes with different composition and microstructure in rabbit femoral condyle model. J Biomed Mater Res B Appl Biomater.

[46] Bohner M, Santoni BLG, Döbelin N (2020). β-tricalcium phosphate for bone substitution: synthesis and properties. Acta Biomater.

[47] Kakuta A, Tanaka T, Chazono M (2019). Effects of micro-porosity and local BMP-2 administration on bioresorption of β-TCP and new bone formation. Biomaterials Res.

[48] Duan R, Barbieri D, De Groot F (2018). Modulating bone regeneration in rabbit condyle defects with three surface-structured tricalcium phosphate ceramics. ACS Biomater Sci Eng.

[49] Davison N, Luo X, Schoenmaker T (2014). Submicron-scale surface architecture of tricalcium phosphate directs osteogenesis in vitro and in vivo. Eur Cell Mater.

[50] Feng B, Jinkang Z, Zhen W (2011). The effect of pore size on tissue ingrowth and neovascularization in porous bioceramics of controlled architecture in vivo. Biomed Mater.

[51] Akao M, Aoki H, Kato K (1982). Dense polycrystalline β-tricalcium phosphate for prosthetic applications. J Mater Sci.

