# Supplementary material for: Improvement of absorbability, osteoconductivity, and strength of a β-tricalcium phosphate spacer for opening wedge high tibial osteotomy: clinical evaluations with 106 patients
Source: BMC Musculoskelet Disord. 2024 Jun 5;25:441. doi: 10.1186/s12891-024-07533-8 (PMC11151500; doi:10.1186/s12891-024-07533-8)
Supplement: Supplementary file 1 — Supplementary Material 1 [file 12891_2024_7533_MOESM1_ESM.pdf]

臨床研究等提出・公開システム

ログアウト

臨床研究・治験計画情報の詳細情報です。

**研究の種別**	非特定臨床研究
**初回公表日**	令和5年6月27日
**最終公表日**	
**中止年月日**	
**観察期間終了日**	
**研究名称**	Improvement of Absorbability, Osteoconductivity, and Strength of a β-Tricalcium Phosphate Spacer for Opening Wedge High Tibial Osteotomy: Clinical Evaluations with 106 Patients
**平易な研究名称**	Tricalcium Phosphate Spacer for Opening Wedge High Tibial Osteotomy
**研究責任（代表）医師の氏名**	近藤　英司
**研究責任（代表）医師の所属機関**	北海道大学病院
**研究・治験の目的**	内側開大式高位脛骨骨切り術に用いる新規スペーサーの有用性を明らかにすること
**試験のフェーズ**	N/A
**対象疾患名**	変形性膝関節症
**進捗状況**	募集中
**医薬品等の一般名称**	
**販売名**	
**認定委員会の名称**	八木整形外科病院倫理委員会
**認定番号**	H29-0002

管理的事項

1　臨床研究の実施体制に関する事項及び臨床研究を行う施設の構造設備に関する事項

2　臨床研究の目的及び内容並びにこれに用いる医薬品等の概要

3　臨床研究の実施状況の確認に関する事項

4　臨床研究の対象者に健康被害が生じた場合の補償及び医療の提供に関する事項

5　臨床研究に用いる医薬品等の製造販売をし、又はしようとする医薬品等製造販売業者及びその特殊関係者の当該臨床研究に対する関与に関する事項等

6　審査意見業務を行う認定臨床研究審査委員会の名称等

7　その他の事項

添付書類（実施計画届出時の添付書類）

閉じる　トップ画面

Copyright　© National Institute of Public Health, All Rights Reserved.
